# Recommendations of the Moroccan Society of Rheumatology (SMR) for the Therapeutic Management of Spondyloarthritis (SpA) including Psoriatic Arthritis (PsA)

**DOI:** 10.31138/mjr.34.2.139

**Published:** 2023-06-30

**Authors:** Nada Jaouad, Lamia Oulkadi, Ibtissam Bentaleb, Ahmed Bezza, Abdellah El Maghraoui, Redouane Niamane, Imane El Bouchti, Saloua Larhrissi, Linda Ichchou, Saadia Janani, Fatima Ezzahra Abourazzak, Nessrine Akasbi, Mariam Erraoui, Samia Karkouri, Ihsane Hmamouchi, Rachid Bahiri

**Affiliations:** 1 Department of Rheumatology A, El Ayachi Hospital, Ibn Sina University Hospital, Salé, Morocco,; 2 Department of Rheumatology, Military Hospital Mohammed V, Ibn Sina University Hospital, Rabat, Morocco,; 3 Private Medical Office of Rheumatology, Rabat, Morocco,; 4 Mohamed V University, Rabat, Morocco,; 5 Department of Rheumatology, Military Hospital Avicenne, Mohammed VI University Hospital, Marrakech, Morocco,; 6 Department of Rheumatology, Arrazi University Hospital, Marrakech, Morocco,; 7 Private Medical Office, Rabat, Morocco,; 8 Department of Rheumatology, Mohammed VI University Hospital, Oujda, Morocco,; 9 Department of Rheumatology, Ibn Rochd University Hospital, Casablanca, Morocco,; 10 Department of Rheumatology, Tanger-Tetouan- Al Hoceima University Hospital, Tanger, Morocco,; 11 Department of Rheumatology, Hassan II University Hospital, Fez, Morocco,; 12 Department of Rheumatology, Hassan II Hospital, CHU Souss Massa, Agadir, Morocco,; 13 CARBONE Research Team, LARISS Laboratory, FMPA, Ibn Zohr University, Agadir, Morocco,; 14 Department of Physical Medicine and Rehabilitation, El Ayachi Hospital, Ibn Sina University Hospital, Salé, Morocco,; 15 Laboratory of Clinical Research and Epidemiology, Faculty of Medicine, Mohammed V University, Rabat, Morocco,; 16 Department of Medicine, Health Science College, International University of Rabat (UIR), Rabat, Morocco

**Keywords:** spondyloarthritis, recommendations, guidelines, treatment, Morocco

## Abstract

The advent of new therapeutic classes and the updating of international recommendations have justified the development of recent recommendations by the Moroccan Society of Rheumatology. Methods Guidelines were drafted by a core steering committee after performing a literature search. A multidisciplinary task force, including three fellows, eleven rheumatologists, a specialist in physical medicine and rehabilitation, an epidemiologist from hospital-university, hospital and liberal sectors and one patient assessed the Best Practice Guidelines using 2 rounds of anonymous online voting by modified Delphi process. Thus, 19 recommendations were developed. Recommendation 1 concerns the therapeutic principles, recommendation 2 insists on the information and education of the patient, recommendation 3 concerns the general measures to be adopted, namely physical activity, smoking cessation and psychological support, recommendation 4 concerns Non-Steroidal Anti-Inflammatory Drugs which constitute the first-line treatment, recommendations 5 to 7 concern the use of analgesics, of general and local corticosteroid therapy and conventional synthetic disease-modifying antirheumatic drugs, recommendations 8 to 13 deal with the use of biologic agents, including new classes and their indications in radiographic and nonradiographic axial and peripheral spondyloarthritis, follow-up and management in case of failure or remission, recommendation 14 deals with the indication for Janus kinase inhibitors drugs, recommendation 15 deals with physical treatment and recommendation 16 deals with the indication of surgery. Recommendations 17 to 19 deal with special situations, namely fibromyalgia, vaccination and pregnancy. A well-defined therapeutic strategy with first- and second-line treatments has been established.

## INTRODUCTION

Spondyloarthritis (SpA) is a heterogeneous disease that can adopt several phenotypic forms. New data in the therapeutic field of axial SpA (axSpA) including non-radiographic axSpA and psoriatic arthritis (PsA) are available. Non-radiographic axSpA can be considered as a recent form of SpA, but its subsequent progression to a radiographic form is not mandatory. Their management is based on the same therapeutic principles as radiographic SpA, although the definition of activity can be complex. Non-radiographic forms without objective signs of inflammation (magnetic resonance imaging MRI or C-reactive protein CRP) have no more therapeutic response with tumour necrosis factor inhibitor (TNFi) than with placebo.^1^

The Moroccan Society of Rheumatology (SMR) had developed in 2017 recommendations for the practical management of patients with SpA.^2^ The advent of new therapeutic classes and the updating of international recommendations have justified the development of recent recommendations by the SMR. The emergence of biotherapies has opened up important therapeutic perspectives. However, they add an additional burden to the socio-economic situation of the disease in our context. These recommendations are based on recent therapeutic data, on the opinion of a multidisciplinary group of professionals and on the latest recommendations.^3,4^ They are intended for all healthcare professionals involved in the management of patients with SpA, and mainly for rheumatologists, in order to make this management consensual.

## METHODS

### Steering committee

The convenor invited a steering committee to develop new SMR recommendations for the management of patients with SpA. Three fellows performed the Systematic Literature Review (SLR). The convenor, the expert rheumatologist and methodologist who had experience of the development of SMR recommendations supervised the fellows’ SLR work. The steering committee included eleven rheumatologists, a specialist in physical medicine and rehabilitation, an epidemiologist, and one patient representative. The SLRs focused on phenotypic management of the disease with successive steps according to the response to treatment. Efficacy of Non-Steroidal Anti-Inflammatory Drugs (NSAIDs), glucocorticoids (GC) and Disease-modifying antirheumatic drugs (DMARDs) including conventional synthetic DMARD (csDMARDs), biological DMARD (bDMARDs), and Targeted synthetic DMARDs (tsDMARDs) are included. The SLRs also focus on non-pharmacological measures. A systematic analysis of the literature published was performed. The SLR has approached registry data, randomised controlled trials and extension studies, recent EULAR and ACR Congress abstracts and recent recommendations through December 2021. Further discussion will focus on the SLR data and suggestions from the steering committee, in order to develop the updated recommendations and voting.

### Delphi process

Two weeks before the first voting round, there was a mail-briefing session where the Taskforce (TF) learned about the decision rules and the level of evidence. The level of agreement of TF members was reported during each round of voting, using a numerical rating scale of one (totally disagree) to nine (totally agree). Participants provided also qualitative comments. The two Delphi rounds were performed through live online meetings moderated by the methodologist of the steering committee. All the members of TF have discussed results during each round. The OMERACT recommendations served as a guide to move from one Delphi round to another and for selecting the final statement.^5^ The consensus was accepted if >80% of the members voted in favour of the recommendation at the first round, second round was accepted if the score > 80% high agreement (7–9) and < 15% low agreement (1–3).

**Figure 1.**
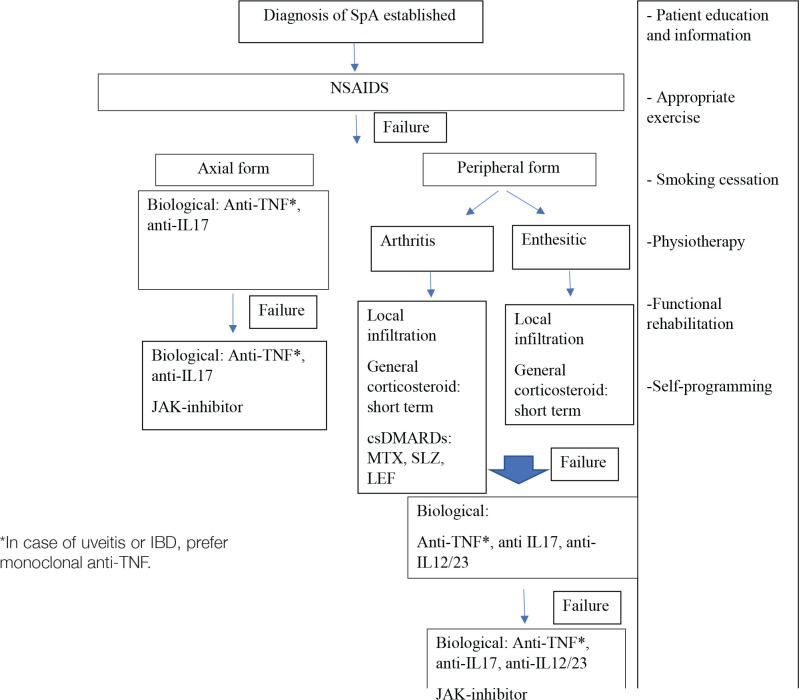
SpA treatment algorithm.

## RESULTS

### Recommendation 1: Principle of treatment

*The management of spondyloarthritis must be early and multidisciplinary, including:*

*-Information and education of the patient*
*-Non-medical treatment*
*-Medical treatment*
*-Management of comorbidities and extra-musculoskeletal manifestations*

Spondyloarthritis management must be global, based on early diagnosis, patient education, and a combination of non-drug and drug treatments. These treatments are complementary and will be adapted to the patients’ comorbidities and expectations.

### Recommendation 2: Patient information and education

*- Patient information and education must be an integral part of care in order to improve patients’ knowledge for a better management of their disease and treatment.*
*- By relying on group and/or individual sessions, therapeutic education allows the patient to communicate with his/her attending physician, to acquire knowledge about his/her disease, to better adhere to treatment, to develop know-how in the face of complications and to adapt on a socio-professional level.*
*- The patient must take a full part in the decision-making process (shared medical decision). Patient associations can also play an important role.*

The patient must take an active role in learning about their disease and in the shared decision-making process. Fayet et al. have shown that patients who have received therapeutic education sessions have good adherence to anti-TNF drugs, particularly if they have received individual sessions.^6^ Mobile health technologies can help the patient to take a role in their disease and its self-management.^7^

### Recommendation 3: General measures

*- Regular and appropriate physical activity should be encouraged.*
*- Smoking must be systematically stopped.*
*-The psychological impact of spondyloarthritis must be managed. The intervention of specialists (psychologist and/or psychiatrist) must be discussed between the rheumatologist and the patient.*

Physical activity has positive benefits on the disease and its comorbidities. It helps to combat stiffness, improve posture, and increase respiratory capacity. It must be adapted to the patient’s profile and comorbidities. The variety of exercise protocols makes it difficult to draw firm conclusions about the most effective exercise prescription. The role of biomechanical stress and microdamage at entheseal sites in the development of tissue specific inflammation and subsequent new bone formation has been discussed.^8^ As a result, the nature of the movement must be considered. Although the exercise protocol is unknown, studies have shown that adding aerobic exercise to traditional stretching and mobility home exercise programs results in superior functional fitness.^9^

Despite its harmful effects, smoking was found in 29% of patients in the ASAS-COMOSPA study.^10^ The different studies have shown that patients who smoke have an early onset of the disease and higher spinal pain, disease activity, functional impact and radiographic progression compared with non-smokers, as well as a lower response to anti-TNF drugs.^11^ Similarly, smoking increases cardiovascular risk and may worsen cardiovascular and pulmonary comorbidities.

The psychological impact must be managed in patients with SpA. Disease has an impact on every part of the patient’s life; thus, the rheumatologist should do a thorough evaluation of mental health. In Europe, 60.7% of patients with axSpA had a risk of mental disturbance, with disease activity being the main contributing factor.^12^ The rheumatologist should regularly evaluate disease activity and early recommend patients to specialists.

### Recommendation 4: NSAIDS

*- NSAIDs are the first-line treatment for SpA, in the absence of contraindications.*
*- Failure to respond to NSAIDs will be considered only after failure of at least 2 different NSAIDs taken at the maximum tolerated dose for a minimum of 2 weeks each.*
*- The duration of treatment is based on the course of the disease. Given the risk of side effects, particularly cardiovascular, gastrointestinal, and renal, continuous use is not recommended if the disease is controlled.*
*- In case of prolonged use, particular vigilance must be maintained regarding their tolerance.*

NSAIDs are the first-line treatment in the absence of contraindications for the rheumatological manifestations of SpA.^13^ There is no significant difference in efficacy between the different classes of NSAIDs, and between radiographic and non-radiographic axSpA.^14^ Naproxen may be preferred in case of cardiovascular risk factors or a selective cyclooxygenase inhibitor in gastrointestinal problems. A gastroprotective agent may be used in combination. Continued treatment in stable SpA is not recommended.^3^ Despite some studies that have demonstrated structural efficacy with continuous use or high doses of NSAIDs, particularly in cases of high CRP,^15^ the assessment of the benefit/risk balance, particularly with regard to cardiovascular, renal, and gastrointestinal risks, does not justify continuous use. The minimum effective dose to control symptoms should be sought.

### Recommendation 5: Analgesics

*- Analgesics can be used for residual pain in combination with other treatments.*

Analgesics can be used for residual pain in combination or in case of contraindication, failure, or intolerance to NSAIDs. There is no new information in this area.

### Recommendation 6: corticosteroid therapy

*- General corticosteroid therapy is not recommended in the axial form; it may be used in cases of unsatisfactory control of peripheral joint manifestations and in the absence of therapeutic alternatives. It must be limited to short periods.*
*- Local corticosteroid injections at symptomatic sites (sacroiliac joints, peripheral joints, and entheses) may be considered.*
*-Topical steroids are the first line treatment in uveitis associated with SpA. Glucocorticoids are also used for moderate-to-severe IBD, but their use is constrained by a number of significant side effects.*

Systemic corticosteroid therapy can be used for resistant peripheral joint manifestations but should be limited to short periods and minimal effective dosages. There is some evidence to suggest that short-term high-dose glucocorticoids may have an effect on axial involvement. However, in the absence of sufficient data and because of the severity of its adverse effects, it is generally not justified for the treatment of axial manifestations.

The efficacy of corticosteroid injection of the sacroiliac joints has been demonstrated in two small, controlled studies with a total of 30 patients and in prospective studies on pain and on the inflammatory signal of the sacroiliac joints on MRI with a duration of efficacy of 6 months of injection. There is no consensus on the injection technique, or the type of corticosteroid injected.^16^

The use of corticosteroids extends to extra-musculoskeletal manifestations such uveitis and IBD. In the majority of cases, topical corticoid is able to control the inflammation of the eye. Systemic therapy of steroids may be indicated in refractory cases and gradually tapering. In IBD, GCs remain the primary treatment for moderate-to-severe disease, but their use is limited by a number of serious side effects. Conventional corticosteroids can be rectally administered to patients with IBD that affects the rectum or the terminal segment of the colon.

### Recommendation 7: csDMARDs

*- The use of csDMARDs (sulfasalazine, methotrexate, leflunomide) should be considered for peripheral and extra-musculoskeletal manifestations (PsA, uveitis, psoriasis, chronic inflammatory bowel disease).*
*- They are not indicated for isolated axial or enthesitic manifestations.*

csDMARDs can be used in refractory peripheral forms and in peripheral PsA. Their indication is also extended to extra-musculoskeletal manifestations. The choice of molecule will depend on the patient’s profile, with a preferential choice of methotrexate in cases of psoriatic skin involvement.^4^ In IBD, methotrexate monotherapy was not superior to placebo for the induction of clinical remission in Crohn’s disease. However, methotrexate was superior to placebo in maintaining clinical remission of Crohn’s disease. Also, methotrexate has not been proven to have efficacy in inducing remission in ulcerative colitis.^17^ No efficacy has been proven in isolated axial or enthesitic forms.

### Recommendation 8: Biologic therapy: Initiation of biologic therapy

*- The initiation of biologic therapy or its biosimilar in axial forms should be proposed to patients whose disease is active and refractory to NSAIDs.*
*- In non-radiographic axSpA, the existence of a biological inflammatory syndrome and/or MRI inflammation is required before initiating a biologic.*
*- The initiation of a biologic treatment or its biosimilar in peripheral forms should be proposed to patients whose disease is active and resistant to conventional treatments (NSAIDs, csDMARDs).*
*- The introduction of a biological treatment may be considered by the expert rheumatologist in other specific situations, for example: refractory coxitis, refractory and/or recurrent uveitis.*

The initiation of biologic therapy in axial forms should be proposed to patients whose disease is active and resistant or intolerant to NSAIDs. NSAID failure is defined as persistence of spinal pain or high activity level after at least 2 different NSAIDs taken at the maximum tolerated dose for a minimum of 2 weeks each. The absence of therapeutic response is also expressed by activity markers: Ankylosing Spondylitis Disease Activity Score (ASDAS) ≥2.1 or Bath Ankylosing Spondylitis Disease Activity Index (BASDAI) ≥4. The finding of high activity should not be limited to a single evaluation. The presence of fibromyalgia may cause an error in the assessment of activity especially in non-radiographic SpA. As a result, objective evidence of activity is required in non-radiographic forms namely biological inflammation without other causes and/or MRI inflammatory sacroiliitis before initiating biological therapy. This restriction avoids overprescribing biologics where the percentage of response will be lower.^1^

The initiation of biologic therapy in peripheral forms should be proposed to patients whose disease is active and resistant despite conventional therapy (NSAIDs ± infiltration and/or at least one csDMARDs in the peripheral joint form and NSAIDs ± infiltration in the peripheral enthesitic form).

Other specific situations may require introduction of biological treatment notably refractory coxitis and refractory and/or recurrent uveitis.

Kui Zhang et al. demonstrated the efficacy of TNFi drugs on hip involvement at the clinical level with improvement in function and on MRI assessed by the Hip Inflammation MRI Scoring System (HIMRISS) at 52 weeks versus control group.^18^

### Recommendation 9: Biologic therapy: Choice of first-line biological treatment for axial/peripheral forms

*- The choice of first-line biological treatment in axial forms (anti-TNF or anti-IL17, generally anti-TNF) must take into account the presence or absence of uveitis or Inflammatory bowel disease (IBD), patient’s profile, and the decision must be shared between the patient and the physician.*
*- The choice of first-line biological treatment in peripheral forms (anti-TNF, anti-IL17, anti-IL23, generally anti-TNF) must take into account the form of SpA (PsA), the presence or absence of uveitis or IBD, patient’s profile, and the decision must be shared between the patient and the physician.*

The choice of first biologic will depend on the disease phenotype, extra-rheumatologic manifestations, comorbidities, contraindications, and the patient’s expectations as part of a shared decision. Current approach is to initiate an anti-TNF due to recoil and the latter’s experience throughout the years, as well as the lack of face-to-face studies in the axial SpA. There do not appear to be any major differences in the efficacy of the various anti-TNF agents.^19^ Monoclonal TNFi are preferred for the treatment of SpA in patients with uveitis or inflammatory enterocolopathies. In the case of a history of uveitis, patients on etanercept had a higher rate of uveitis flares compared to pre-treatment (60 vs 42), in contrast to adalimumab and infliximab, which had a lower rate of uveitis (14 vs 37 for adalimumab, 28 vs 46 for infliximab).^20^ Also, patients with SpA who receive infliximab or adalimumab have a lower risk of IBD exacerbation than those who receive etanercept.^3^ These data suggest that etanercept has less of a protective effect against previous IBD or uveitis. Regarding anti-TNF agents, 14/17 of RCTs provided evidence of efficacy in achieving remission at different time: 12, 16, 24 and 28 weeks (ASAS-PR in 16–62% of patients and ASDAS-ID in 24–40% of patients). With a limited number of studies available, IL-17A inhibitors showed remission rates of 15–21% for ASAS-PR and 11–16% for ASDAS-ID at week 16.^21^ In the 5-year extension of the MEASURE-1 study, secukinumab demonstrated efficacy on clinical symptoms, function, and objective markers of inflammation with a good safety profile over 5 years.^22^ For the non-radiographic axial form, secukinumab 150 mg provided significant improvement in clinical symptoms through week 52 and also on bone oedema on MRI with a good safety profile.^23^ For PsA, two head-to-head studies found a similar efficacy and safety profile comparing anti-TNF to IL-17.^24,25^ Anti-IL17 or IL-12/23 may be preferred in cases of skin involvement. In cases of associated skin psoriasis, the dose of anti-IL-17 may be increased.

**Table 1.** Indication of a biological treatment.

**Radiographic axial SpA**	**Non-radiographic axial SpA**	**Peripheral joint SpA**	**Peripheral enthesitic SpA**
Insufficient NSAID response + BASDAI ≥ 4 or ASDAS ≥ 2.1	Insufficient NSAID response + BASDAI ≥ 4 or ASDAS ≥ 2.1 + Positive CRP and/or inflammation MRI	Insufficient NSAID response ± infiltration + ≥ 1 DMARD + NAG and NAD* ≥ 3	Insufficient NSAID response and ± infiltration + Objective signs of inflammation Pain ≥ 4

* Lower number if coxitis or arthritis refractory to infiltration or radiographic progression.

NAG: number of swollen joints. NAD: number of tender joints.

BASDAI, ASDAS, NAG, NAD, and CRP: found at 2 visits one month apart.

### Recommendation 10: Biologic therapy: Pre-therapeutic assessment

*- The prescription of a biological treatment requires a pre-therapeutic assessment to identify any absolute or relative contraindications, to detect comorbidities and to limit the risk of infection.*
*- This assessment will help the rheumatologist to prescribe most appropriate biological treatment to the patient’s profile and the disease.*

Pre-therapeutic assessment help to limit the risk of infection and to screen for contraindications. Particular attention is paid to tuberculosis in our context. In the Moroccan biotherapy registry, the prevalence of latent tuberculosis before the initiation of biotherapy in spondyloarthritis was 24.8%.^26^ Chemoprophylaxis is necessary to avoid the risk of tuberculosis reactivation which may be increased in this context. Based on national recommendations, before beginning a biological treatment, patients who have undergone testing for latent tuberculosis must receive preventive care. Tuberculin skin test, an IGRA test, or both tests are used for screening latent tuberculosis. Typically, the treatment scheme relies on isoniazid for 6 or 9 months or isoniazid-rifampicin for 3 months, depending on the patient’s history.^27^

### Recommendation 11: Biologic therapy: Switching of biologics

*- In the case of loss of response, primary inefficacy, or intolerance to a first anti-TNF in axial forms, switching to a second anti-TNFα agent or a non-anti-TNF biologic such as secukinumab or ixekizumab (anti-IL17) may be considered.*
*- In case of loss of response, primary inefficacy, or intolerance to a first anti-TNF in peripheral forms, switching to a second anti-TNFα agent or a non-anti-TNF biologic agent such as secukinumab or ixekizumab (anti-IL17) or ustekinumab (anti-IL-12/23) may be considered.*

In case of loss of response, primary inefficacy, or intolerance to a first biologic, switching from one anti-TNF to a second anti-TNF agent is possible with comparable efficacy or a change of class can be considered.^28^

### Recommendation 12: Biologic therapy: Evaluation of therapeutic response

*- The response to biological treatments will be evaluated 12 weeks after their initiation using activity criteria adapted to the phenotypic form.*

The evaluation of the therapeutic response must be done by composite scores adapted to each form. Inactive disease, particularly in the axial form, can be defined by an ASDAS score lower than 1,3. The scores derived from the DAS are preferred in forms with predominantly peripheral involvement. In PsA, the Disease activity index for PsA (DAPSA) may be preferred in practice.^29^ Assessment of therapeutic response should be done 12 weeks after initiation of biologic therapy. The therapeutic objective should also include the reduction of NSAID, the activity of extra-musculoskeletal manifestations and the structural evolution.

### Recommendation 13: Biologic therapy: In case of prolonged remission

*- In case of remission or low activity maintained for at least 6 months on biologics, progressive spacing of administrations or reduction of treatment dosage should be considered. Spacing is currently more recommended.*
*- It is generally preferable to continue the biological treatment, as this exposes to a lower risk of relapse compared to stopping the treatment.*

Data from the literature are currently available to assess progressive spacing of administrations or reduction of treatment in case of achieving remission or low disease activity (LDA). Spacing study is a non-inferiority randomized controlled trial in patients with LDA for at least 6 months. Among 373 patients with complete follow-up (93.7%), 162/184 (88.0%) had LDA in the “space” group compared with 173/189 (91.5%) in the “unchanged” group at 12 months.^30^ In the event of relapse after attempted spacing, resumption of treatment at the initial dosage and frequency would, in most cases, restore the previous response. Sudden discontinuation may be accompanied by a relapse of the disease. COAST-Y study and SPIRIT-P3 study evaluate the effect of continuing versus withdrawing ixekizumab in patients respectively with axSpA and PsA. Continued treatment was associated with a lower risk of relapse compared to discontinuation.^31,32^ According to a study on flare frequency dependent on dose reduction, 99% of axSpA patients in clinical remission flared during tapering to discontinuation, whereas more than half had not receive before 1/3 dose or less before.^33^

### Recommendation 14: tsDMARDs

*- JAK inhibitors can be used in axial forms or in peripheral forms with or without psoriasis not responding to at least one bDMARD.*

Three JAKi have been evaluated in radiographic axSpA. Most patients were bDMARD naïve. SELECT-AXIS2 (study 2) evaluated the efficacy and safety of upadacitinib compared to placebo in patients with non-radiographic axSpA. Upadacitinib achieved the ASAS40 primary endpoint at week 14 against placebo (45% versus 23%) in the first outcome.^34^ Tofacitinib was effective in a randomized, double-blind, phase III, placebo-controlled study at a dose of 5mg twice daily on activity parameters with a good safety profile in patients who had failed or were intolerant to at least two NSAIDs.^35^ A phase 2 trial (TORTUGA) showed that filgotinib 200mg/d for 12 weeks was effective in SpA compared to placebo.^36^ It also demonstrated a decrease in MRI inflammatory activity on spinal lesions and posterolateral and facet elements at 12 weeks.^37^ There was not a higher rate of extra-musculoskeletal manifestations with JAKi compared to placebo but more data are necessary specially to evaluate the impact of JAKi in acute uveitis or on flares of uveitis. JAKi can be use in the treatment of peripheral arthritis in PsA.^4^ It has shown a significantly higher ACR20 response rate compared to placebo in 2 phase III studies on Tofacitinib, 1 phase II study on Filgotinib and 2 phase III studies on Upadacitinib without significant higher risk of serious adverse events.^38^ It also can improve psoriasis lesions. Overall, JAK inhibitors represent an interesting therapeutic option with positive outcomes in SpA and PsA. However, the greater data basis and longer experience with anti-TNF and recently with IL-17 makes it difficult to prioritize or put in the same line with bDMARDs. Following the FDA warning on serious side effects namely risk of thrombosis, infection and heart attack, the safety profile at long-term of JAKi is still debated specially in patients with multimorbidity.

### Recommendation 15: Rehabilitation and equipment

*- Rehabilitation sessions with an adapted self-program should be proposed.*
*- Hip rehabilitation in case of coxitis should be offered at an early stage in order to maintain a better autonomy and quality of life.*
*- In some situations, orthoses and corsets are necessary to prevent and stop possible deformities.*

Rehabilitation must be encouraged and started as soon as the diagnosis is made. It can be carried out in individual or group sessions. Therapeutic education is an important parameter in this case for a better adherence of the patients. Several therapies have been proposed in the literature.^39^ The approach must include teaching of postures, stretching work, in particular postural stretching, muscle strengthening and respiratory work. A self-program, first supervised and then carried out by the patient at home or with the help of video-internet, should be recommended with a variability of exercises to avoid monotony. Analgesic therapies can be proposed, notably cryotherapy, physiotherapy and balneotherapy.

**Table 2.** SMR recommendations for the therapeutic management of Spondyloarthritis including Psoriatic Arthritis with level of agreement.

**Recommendations**	**Level of agreement, mean (SD)**
R1: Principle of treatment The management of spondyloarthritis must be early and multidisciplinary, including: -Information and education of the patient -Non-medicinal treatment -Medicinal treatment -Management of comorbidities and extra-rheumatological manifestations	9 (0,0)
R2: Patient information and education - Patient information and education must be an integral part of care in order to improve patients’ knowledge for better management of their disease and treatment. - By relying on group and/or individual sessions, therapeutic education allows the patient to communicate with his/her attending physician, to acquire knowledge about his/her disease, to better adhere to treatment, to develop know-how in the face of complications and to adapt on a socio-professional level. - The patient must take a full part in the decision-making process (shared medical decision). Patient associations can also play an important role.	9 (0,0)
R3: General measures - Regular and appropriate physical activity should be encouraged. - Smoking must be systematically stopped. -The psychological impact of spondyloarthritis must be managed. The intervention of specialists (psychologist and/or psychiatrist) must be discussed between the rheumatologist and the patient.	8,8 (0,5)
R4: NSAIDS - NSAIDs are the first-line treatment for SpA, in the absence of contraindications. - Failure to respond to NSAIDs will be considered only after failure of at least 2 different NSAIDs taken at the maximum tolerated dose for a minimum of 2 weeks each. - The duration of treatment is based on the course of the disease. Given the risk of side effects, particularly cardiovascular, gastrointestinal and renal, continuous use is not recommended if the disease is controlled. - In case of prolonged use, particular vigilance must be maintained regarding their tolerance.	8,8 (0,4)
R5: Analgesics -Analgesics can be used for residual pain in combination with other treatments.	8,7 (0,6)
R6: Corticosteroid therapy - General corticosteroid therapy is not recommended in the axial form; it may be used in cases of unsatisfactory control of peripheral joint manifestations and in the absence of therapeutic alternatives. It must be limited to short periods. - Local corticosteroid injections at symptomatic sites (sacroiliac joints, peripheral joints, and entheses) may be considered. -Topical steroids are the first line treatment in uveitis associated with SpA. Glucocorticoids are also used for moderate-to-severe IBD, but their use is constrained by a number of significant side effects.	8,8 (0,4)
R7: csDMARDs - The use of csDMARDs (sulfasalazine, methotrexate, leflunomide) should be considered for peripheral and extra-rheumatological forms (PsA, uveitis, psoriasis, chronic inflammatory bowel disease). - They are not indicated for isolated axial or enthesitic manifestations.	8,8 (0,3)
R8: Biologic therapy: initiation of biologic therapy - The initiation of biologic therapy or its biosimilar in axial forms should be proposed to patients whose disease is active and refractory to NSAIDs. - In non-radiographic axSpA, the existence of a biological inflammatory syndrome and/or MRI inflammation is required before initiating a biologic. - The initiation of a biologic treatment or its biosimilar in peripheral forms should be proposed to patients whose disease is active and resistant to conventional treatments (NSAIDs, csDMARDs). - The introduction of a biological treatment may be considered by the expert rheumatologist in other specific situations, for example: refractory coxitis, refractory and/or recurrent uveitis.	8 (1,3)
R9: Biologic therapy: choice of first-line biological treatment for axial/peripheral forms - The choice of first-line biological treatment in axial forms (anti-TNF or anti-IL17, generally anti-TNF) must take into account the presence or absence of uveitis or Inflammatory bowel disease (IBD), patient’s profile, and the decision must be shared between the patient and the physician. - The choice of first-line biological treatment in peripheral forms (anti-TNF, anti-IL17, anti-IL23, generally anti-TNF) must take into account the form of SpA (PsA), the presence or absence of uveitis or IBD, patient’s profile, and the decision must be shared between the patient and the physician.	8,7 (0,6)
R10: Biologic therapy: pre-therapeutic assessment - The prescription of a biological treatment requires a pre-therapeutic assessment to identify any absolute or relative contraindications, to detect comorbidities and to limit the risk of infection. - This assessment will help the rheumatologist to prescribe most appropriate biological treatment to the patient’s profile and the disease.	8,7 (0,6)
R11: Biologic Therapy: switching of biologics - In the case of loss of response, primary inefficacy, or intolerance to a first anti-TNF in axial forms, switching to a second anti-TNFα agent or a non-anti-TNF biologic such as secukinumab or ixekizumab (anti-IL17) may be considered. - In case of loss of response, primary inefficacy or intolerance to a first anti-TNF in peripheral forms, switching to a second anti-TNFα agent or a non-anti-TNF biologic agent such as secukinumab or ixekizumab (anti-IL17) or ustekinumab (anti-IL-12/23) may be considered.	8,5 (0,7)
R12: Biologic therapy: evaluation of therapeutic response - The response to biological treatments will be evaluated 12 weeks after their initiation using activity criteria adapted to the phenotypic form.	8,8 (0,3)
R13: Biologic therapy: in case of prolonged remission - In case of remission or low activity maintained for at least 6 months on biologics, progressive spacing of administrations or reduction of treatment dosage should be considered. Spacing is currently more recommended. - It is generally preferable to continue the biological treatment, as this exposes to a lower risk of relapse compared to stopping the treatment.	8,7 (0,4)
R14: tsDMARDs - JAK inhibitors can be used in axial forms or in peripheral forms with or without psoriasis not responding to at least one bDMARD.	8,9 (0,2)
R15: Rehabilitation and equipment - Rehabilitation sessions with an adapted self-program should be proposed. - Hip rehabilitation in case of coxitis should be offered at an early stage in order to maintain a better autonomy and quality of life. - In some situations, orthoses and corsets are necessary to prevent and stop possible deformities.	8,7 (0,4)
R16: Surgical treatment - The indication for surgery must be determined on a case-by-case basis (destructive peripheral arthritis, spinal ankylosis with major deformity or spinal fracture), taking into account pain, functional incapacity under treatment, structural damage, the terrain/comorbidities and the patient’s wishes and expectations.	8,8 (0,3)
R17: Fibromyalgia - The association of SpA and fibromyalgia is frequent. - It may interfere with the evaluation of the disease activity and may lead to inappropriate prescription of biological treatments. - Rapid detection must be systematic. Validated questionnaires such as FIRST can be used. - The management of fibromyalgia must be adapted and personalized, giving priority to non-drug treatments.	8,5 (0,7)
R18: Vaccination and SpA - It is recommended that patients with SpA have their vaccinal status checked. - It is recommended that the vaccination calendar must be updated, particularly against influenza, pneumococcus and hepatitis B, while respecting the time limit for stopping immunosuppressive treatments in the case of attenuated vaccines. - Vaccination against Covid-19 as well as preventive measures are recommended in patients with SpA to prevent a severe form of covid-19.	8,8 (0,3)
R19: Pregnancy and SpA - The activity of SpA during pregnancy is difficult to assess in the case of axial relapse. Peripheral arthritis and extra-articular manifestations, in particular uveitis, tend to decrease except for ulcerative colitis flare-ups. - The contraindications specific to each drug treatment must be taken into account in case of desire for pregnancy. - Continuation of treatment with salazopyrine (in peripheral forms) or anti-TNF drugs during conception or gestation is possible. Certolizumab is preferred because of its safety. - A study of the benefit/risk ratio as part of a shared decision between the patient and the physician is essential.	8,4 (0,9)

### Recommendation 16: Surgical treatment

*- The indication for surgery must be determined on a case-by-case basis (destructive peripheral arthritis, spinal ankylosis with major deformity or spinal fracture), taking into account pain, functional incapacity under treatment, structural damage, the background/comorbidities and the patient’s wishes and expectations.*

Prosthetic replacement of the hip or knee may be proposed in cases of destructive damage with major functional impairment. Hip replacement surgery is not age-related; thus, it can be done at any age and even earlier if necessary. Spinal surgery in SpA includes osteosynthesis (arthrodesis) to stabilize fractures and osteotomies to correct severe anterior imbalances.^40^ These are difficult surgeries, with specific risks (intubation, vascular and neurological complications).^41^

### Recommendation 17: Fibromyalgia

*- The association of SpA and fibromyalgia is frequent.*
*- It may interfere with the evaluation of the disease activity and may lead to inappropriate prescription of biological treatments.*
*- Rapid detection must be systematic. Validated questionnaires such as FIRST can be used.*
*- The management of fibromyalgia must be adapted and personalized, giving priority to non-drug treatments.*

The prevalence of FM associated with SpA in a meta-analysis of 14 studies is 18%,^42^ vs 2–4% in the general population.^43^ In this cohort, the presence of FM has been shown to impact SpA activity criteria, which may have therapeutic implications. Management may be difficult. From a meta-analysis of 224 trials including a total of 29,962 participants, the authors conclude that treatments for FM are of limited effectiveness and that most currently available treatments for the management of fibromyalgia are not supported by high quality evidence.^44^

### Recommendation 18: Vaccination and SpA

*- It is recommended that patients with SpA have their vaccinal status checked.*
*- It is recommended that the vaccination calendar must be updated, particularly against influenza, pneumococcus, and hepatitis B, while respecting the time limit for stopping immunosuppressive treatments in the case of attenuated vaccines.*
*- Vaccination against Covid-19 as well as preventive measures are recommended in patients with SpA to prevent a severe form of Covid-19.*

Given the high infectious risk in patients with chronic inflammatory rheumatic diseases, and even more with biotherapy,^45^ expert groups have developed updated recommendations for vaccination in chronic inflammatory rheumatic diseases and autoimmune diseases.^46^

Given the current global pandemic context, Moroccan recommendations for good practice concerning vaccination against covid-19 and management of treatments during the vaccination period in patients with chronic inflammatory rheumatic diseases have been developed.^47^

This population may remain under-vaccinated due to a misunderstanding of the attitude to adopt in this context.

### Recommendation 19: Pregnancy and SpA

*- The activity of SpA during pregnancy is difficult to assess in the case of axial relapse. Peripheral arthritis and extra-musculoskeletal manifestations, in particular uveitis, tend to decrease except for ulcerative colitis flare-ups.*
*- The contraindications specific to each drug treatment must be taken into account in case of desire for pregnancy.*
*- Continuation of treatment with salazopyrine (in peripheral forms) or anti-TNF drugs during conception or gestation is possible. Certolizumab is preferred because of its safety.*
*- A study of the benefit/risk ratio as part of a shared decision between the patient and the physician is essential.*

Conception of a pregnancy in SpA should be done in the context of a shared medical decision and taking into account the contraindications of each ongoing treatment. Given the good tolerance of anti-TNF and secukinumab exposure during pregnancy and before conception,^48^ continued treatment with an anti-TNF or anti-IL17 during conception or gestation is possible.^49^

Certolizumab is more preferred as it does not contain an Fc-chain and therefore has minimal placental transfer.^49,50^

## DISCUSSION

The recommendations provide a treatment strategy with successive steps as long as the target is not reached and insist on the principle of phenotypic management of the disease. NSAIDs are the first-line treatment whatever the clinical presentation is.^3^ Conventional disease-modifying therapies are useful in refractory peripheral forms and in peripheral form of PsA if symptomatic treatment fails. Their indication is also extended to extra-musculoskeletal manifestations.^4^ In case of enthesitis and/or dactylitis peripheral form, conventional treatments are not indicated. Targeted therapy should be considered if the disease remains active despite well-conducted NSAID therapy in predominantly axial forms and NSAID and csDMARDs therapy in predominantly peripheral forms. The choice of biological agent must be based on a medical decision shared with the patient and taking into account the extra-musculoskeletal manifestations, co-morbidities and tolerance. Therefore, an anti-TNF should be preferred if IBD or recurrent uveitis is associated.^19,21^

After failure of a first targeted treatment, a change of bDMARD may relate to a different class (for instance, switching from an anti-TNF to an anti-IL-17), or it may involve switching from one anti-TNF to another anti-TNF.^28^ There are no data on the effectiveness of JAK inhibitors in the event that anti-TNF or anti-IL-17 fail. In case of remission or low activity maintained for at least 6 months under biomedication, progressive spacing of administrations or dosage reduction can be considered.^30^

JAK inhibitors offer new treatment options for SpA. In addition to PsA, their use in active axSpA is very promising.^34–37^

The recommendations also focus on non-pharmacological measures. Providing information to the patient and insisting on therapeutic education is required. They also encourage the need of physical activity, smoking cessation and psychological care. Rehabilitation sessions with an adapted self-program should be proposed particularly in axial forms.

We have also included in our recommendations the importance of vaccination and updating of immunization status given the high risk of infection in these patients. This update also describes the approach in case of pregnancy as part of shared decision-making process between patients and their physicians.

## CONCLUSION

This work proposes an update of the recommendations of the SMR for the therapeutic management of patients with SpA in current practice to help meet the needs of patients and clinicians. In addition to new therapeutic options, these recommendations reinforce the phenotypic management of the disease, the shared medical decision, and the non-pharmacological measures.

## ABBREVIATIONS

ASDAS: Ankylosing Spondylitis Disease Activity Score
BASDAI: Bath Ankylosing Spondylitis Disease Activity Index
CRP: C-reactive protein
DAPSA: Disease activity index for PsA
DMARDs: Disease-modifying antirheumatic drugs
GC: Glucocorticoids
IBD: Inflammatory bowel disease
MRI: Magnetic resonance imaging
NSAIDs: Non-Steroidal Anti-Inflammatory Drugs
PsA: Psoriatic arthritis
SLR: Systematic Literature Review
SMR: Moroccan Society of Rheumatology
SpA: Spondyloarthritis
TF: Taskforce
TNFi: Tumour necrosis factor inhibitor
axSpA: Axial spondyloarthritis
bDMARDs: Biological DMARDs
csDMARDs: Conventional synthetic DMARDs
tsDMARDs: Targeted synthetic DMARDs
